# Impact of a clinical care pathway developed through the action research method on the psychological well-being and quality of life in male patients with urethral stricture

**DOI:** 10.1097/MD.0000000000037321

**Published:** 2024-03-01

**Authors:** Yanhong Yan, Yue Wu, Anqi Li, Aiying Yang, Jun Tao, Xuejing Wang

**Affiliations:** a Urology Department, Jiangsu Province Hospital, and the First Affiliated Hospital of Nanjing Medical University, Nanjing, China.

**Keywords:** action research method, anxiety, clinical nursing path, de-pressed, quality of life, urethral stricture

## Abstract

**Background:**

The objective of this study is to examine the development of a clinical care pathway utilizing an action research methodology for male patients with urethral stricture, and to assess the psychological and quality of life outcomes following the implementation of this pathway.

**Methods:**

Ninety patients diagnosed with urethral stricture, admitted to our hospital between May 2021 and May 2022, were selected as the study cohort. Employing a random number method, these patients were allocated into an observation group and a control group, each comprising 45 individuals. The control cohort employs standard care protocols for individuals with urethral stenosis, while the experimental group employs an action research methodology to develop a clinical care pathway specific to the management of patients with urethral stenosis, with an intervention cycle of 3 months. The investigation evaluated the impact of the intervention by scrutinizing pre- and post-intervention data through the utilization of the WHO Quality of Life Scale (WHOQOL-BREF), in addition to the Anxiety Rating Scale and the Depression Rating Scale.

**Results:**

Prior to the intervention, no significant differences were observed in WHOQOL-BREF scores across dimensions, as well as anxiety and depression scores between the 2 groups (*P* > .05). Subsequent to the intervention, the patients in the observation group exhibited significantly higher scores across all WHOQOL-BREF dimensions and total scores compared to the control group, with statistical significance (*P* < .05). Moreover, anxiety and depression scores in the observation group were markedly lower than those in the control group, demonstrating statistical significance (*P* < .05).

**Conclusion:**

The implementation of a clinical nursing pathway rooted in action research methodology proves to be an effective strategy for enhancing clinical nursing practices, elevating patient quality of life, and diminishing the prevalence of anxiety and depression.

## 1. Introduction

Urethral stenosis stands as a prevalent ailment within the realm of urology, predominantly afflicting the male demographic. Characterized by the constriction of the urethral lumen at various segments, this condition precipitates augmented urethral resistance, thereby culminating in obstructed urinary flow^[1]^; the fundamental pathological changes are progressive urethral mucosa and submucosal tissue fibrosis, the mildly stenotic segments are only membranous, and in severe cases, can lead to the complete occlusion of the urethral lumen. The incidence of urethral stenosis shows an increasing trend along with the increase in age; according to statistics, the incidence rate of adult males ranges from 2.2% to 9.8%, and the incidence rate of males older than 65 is even higher.^[2]^

Surgery has become an important treatment modality for urethral stricture, and the most commonly used surgical procedures include transurethral prostatectomy, transurethral resection of the prostate, and open prostatectomy, of which transurethral resection of the prostate is still the gold standard for the treatment of urethral stricture.^[3]^ Urethral stenosis can cause urinary difficulties, urinary retention, and penile erection disorders. It can seriously lead to urinary tract infections or even uremia, which seriously impacts a patient’s quality of life and physical and mental health.^[4]^ Individuals with urethral stenosis frequently experience preoperative challenges, including depression, anxiety, and other adverse reactions attributed to the prolonged symptoms of progressive dysuria, thereby influencing their daily life and interpersonal interactions.^[5]^ The onset of urethral stenosis following transurethral resection of the prostate exacerbates anxiety and depression in elderly patients. This not only significantly impacts their postoperative recuperation but also results in diminished confidence in treatment, rehabilitation, and overall life.^[6]^ Moreover, the prevalence of complications postsurgical intervention significantly impacts the quality of life for both patients and their family members. The physical and mental well-being of patients is substantially jeopardized, exerting a pronounced influence on clinical outcomes. Simultaneously, this places a strain on medical resources and entails high treatment costs, emerging as a prominent societal concern that commands public attention. Researchers have delved into the assessment of patients’ quality of life, with its origins dating back to the 1920s in the United States within the realm of social science research. This area of study garnered substantial attention from medical scholars through the late 1970s. Grounded in the novel medical paradigm that integrates the psychological, biological, and social facets of human existence, the concept of quality of life emerged as intricately linked to health. This holistic perspective has shaped a nuanced understanding of health, transcending traditional biomedical frameworks. In recent years, urologists have dedicated significant research efforts to enhancing the quality of life in patients following urethral stenosis. Treatment programs have shifted focus from disease eradication to implementing measures aimed at alleviating the negative psychological aspects associated with the patient’s condition. The emphasis is on strategies to optimize the patient’s quality of life, recognizing the broader impact of urethral stenosis beyond the primary goal of disease elimination. This approach underscores a comprehensive perspective, acknowledging the importance of addressing psychological well-being in the overall care and outcomes for patients with post-urethral stenosis.

Evidence-based clinical care pathways are both an efficient, standardized model of care based on science and a powerful quality management tool that can effectively improve the quality of care.^[7]^ The action research method is a new educational research method that originated in Western countries in the 1970s. It is a research method that closely combines the problems encountered in practical work and research to improve the quality of action, with the actual work as the primary goal, and the ultimate goal is to solve the actual problem.^[8]^ Action research methodology is based on critical theorizing. It provides continuous and dynamic intervention and quality improvement for practitioners during practice sessions through a cyclical model of identifying a problem, making a plan, taking action, observing the effects, reflecting, and planning again.^[9]^ The action research method, with its extensive applicability across various domains of nursing, has made significant inroads in research, education, and practice, yielding commendable outcomes.^[10]^ Nevertheless, there is a paucity of studies employing the action research method in the development of clinical nursing pathways, particularly in the context of patients with urethral stenosis. Addressing this gap, the current study employs the action research method to construct a clinical nursing pathway tailored for male urethral stenosis patients within our department, resulting in highly favorable outcomes. The ensuing report provides a detailed account of the methods utilized and the noteworthy effects observed.

## 2. Materials and methods

### 2.1. General information

Ninety male urethral stricture patients admitted to our hospital between May 2021 and May 2022 were selected for the study. Inclusion criteria: age > 40 years; meeting the diagnostic criteria in the guidelines for the diagnosis and treatment of urethral strictures published by European Urology^[11]^; proposed surgery; informed consent and willingness of the patient or family. Exclusion criteria: unconsciousness or cognitive dysfunction; suffering from psychiatric diseases; comorbid cardiovascular disease; patients who withdrew or died during the study. This study is a comparison of the means of the 2 samples, so the formula is: $n=\frac{(\sigma_{1}^{2}+\sigma_{2}^{2}/r){\left(u\alpha /2+u\beta \right)}^{2}}{{\left(\mu_{1}-u_{2}\right)}^{2}}$, the results of the review of relevant literature to calculate the total number of samples is 67 cases, taking into account the 10% loss of visits so that the sample size will be expanded to 90 cases. The random number method was used for grouping. There were 45 cases in both the observation group and the control group; comparing the general data of age, education, degree of urethral stenosis, duration of the disease, and surgical methods of the patients in the 2 groups, and the differences were not statistically significant (*P* > .05) (see Table 1). After review and approval by the hospital ethics committee, all patients or family members signed an informed consent form.

**Table 1:** Clinical care pathway for male urethral stricture patients.

Time	Project	Specific measures
Preoperative	Completion of preoperative examinations and preparations, preoperative health education and emotional reassurance	1. Facilitate and execute pertinent examinations (urodynamic examination, cystoscopy urethroscopy, urography, urine routine, and mid-stream urine bacterial culture). 2. Preoperative preparations include the following: for patients necessitating tongue mucosa transplantation, perform oral hygiene practices twice daily; conduct gargling with Yintong gargle post-meals in the morning, midday, and evening for 3 days preceding the operation; administer an enema on the evening before the operation or oral intake of sodium carbonate; implement perineum skin preparation. 3. Preoperative health education entails informing patients and their families about the purpose and significance of preoperative examinations, precautionary measures, cooperation requirements, and anticipated results. This ensures a comprehensive understanding, guiding them through the relevant examinations. Additionally, an overview of the operative environment, anesthesia mode, anesthesia position, surgical procedure mode, and illustrated surgical positioning is provided.
Postoperative	Pain care	Surgical Site Pain Management: Postoperative analgesics were administered epidurally. Assessment of oral pain utilized a visual analogue scale. If the pain score was below 2 points, a cooling patch was applied bilaterally to the cheeks. In cases where the pain score exceeded 2 points, severe instances warranted a 0.5% lidocaine gargle, accompanied by the application of a cooling patch.
	Postoperative tube care	Urinary Catheter Management: Employ Jiyushen perineal care twice daily to proactively prevent urinary tract infections. Vesicostomy Tube Care: Ensure unimpeded drainage of the vesicostomy tube, securely affix it in place, and provide a comprehensive report on color and volume during each shift handover.
	Dietary care	Upon restoration of normal swallowing function, provide guidance to the patient to consume a modest quantity of warm water (utilize cold boiled water for individuals undergoing tongue mucosa transplantation). In the initial 1 to 3 days post-surgery, introduce a diet comprising warm and tepid foods in small portions, gradually progressing to larger meal volumes. Patients who have undergone tongue mucosa transplantation should incorporate fiber-rich foods into their diet once oral wound healing permits, promoting regular bowel movements. Aim for a daily water intake ranging from 2000 to 3000 mL. By the fourth postoperative day, transition to a regular diet, ensuring a daily water intake exceeding 2000 mL.
	Psychological care	Facilitate patients in psychological and emotional regulation. In cases where patients manifest pronounced negative emotions, such as anxiety, depression, and restlessness, prompt psychological counseling is administered. The establishment of a positive nurse-patient rapport is emphasized, incorporating cognitive-behavioral therapy into psychological care. Additionally, guidance is extended to family members on patient care, encouraging active patient engagement with the treatment regimen.
	Educational meetings	Conduct routine postoperative patient education sessions focused on urethral stenosis, elucidating the pertinent risk factors, clinical presentation, treatment modalities, and precautionary measures. Detailed guidance is provided to patients on the proper execution of pelvic floor muscle exercises, with an additional directive for family members to oversee and support the patients in adhering to the prescribed exercises.
	Discharge follow-up guidance	Prior to discharge, patients and their families received the department’s proprietary “Post-discharge Health Education Manual for Urethral Stenosis.” This manual encompassed comprehensive guidance on home care practices, the timing of reviews and follow-ups, and training for restoring urinary function post-urethral catheter removal. Post-discharge, a continuum of care was ensured. Patients or their families were encouraged to join a WeChat group dedicated to urethral stenosis, where inquiries could be addressed promptly. The director, chief nursing officer, and key personnel actively participated in the group, responding to queries and disseminating valuable content through regular live broadcasts. To enhance accountability, family members were entrusted with supervising the patient’s daily pelvic floor muscle function exercise, documented through clock-ins on the WeChat group. Instructional videos were provided to guide corrective actions for any irregular movements.

### 2.2. Research technology roadmap

**Figure 1.**
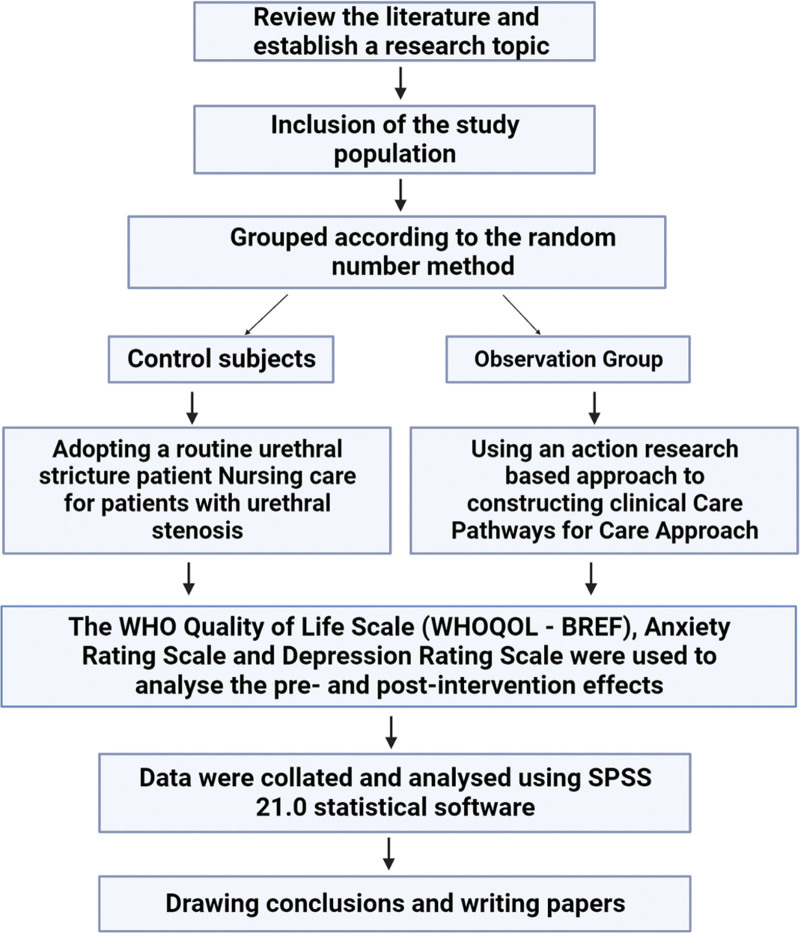
The technical roadmap of this study.

The technical route of this study is shown in Figure 1.

### 2.3. Methods

This experimental study protocol was approved by the Ethics Committee of the First Affiliated Hospital of Nanjing Medical University (LUN Review number: 2021-SRFA-059).

Formation of the Research Team. The intervention team for this study comprised 8 members, including a chief nurse with expertise in urology nursing, a health manager guiding the nursing program using the action research method, a master’s degree student in medicine, a master’s degree student in nursing, and 4 nursing staff members with over 5 years of experience. All members, including the nursing researchers involved in the action research methodology, underwent a comprehensive 2-week training on action research and related knowledge. At the conclusion of the training, an assessment was conducted, and all team members successfully passed the evaluation.

A clinical randomized controlled study was conducted. The control group adopted the routine care of patients with urethral stricture. Preoperative health education informed patients of 12 hours of preoperative fasting and 6 hours of abstinence from drinking. It informed patients of the purpose of the examination and testing and the preparation of intraoperative supplies. Patients were given decubitus lying down with heads tilted to 1 side for 6 hours postoperatively, instructed to pay attention to rest and medication, routine discharge instructions, etc. The observation group was based on the action research method. First, for the department, what are the problems of the current clinical pathway for patients with urethral stenosis? For these problems, through planning, action, observation, reflection, revision of the plan, and implementation, we built a new set of care pathways for patients with urethral stenosis. We applied it to the patients, which was implemented as follows.

1. Raise a question. The head nurse organized a meeting of all nursing staff in the department to analyze the problems of the current nursing pathway for patients with urethral stenosis in the department using semi-structured group interviews, and the nurses unanimously agreed that the current preoperative and postoperative nursing routines for patients with urethral stenosis in our department were rather general and insufficiently detailed, which resulted in nurses not being able to implement the nursing measures in a structured manner. Many of the entries in the nursing routines are outdated and fail to change according to the latest national and international research.
2. Program. The first draft of the clinical care pathway for male urinary tract stenosis was produced by reviewing relevant literature at home and abroad and then combining it with the actual situation of the department. The first draft of the clinical care pathway was solicited from the head of the department, the head nurse and postgraduate nursing students, and also 5 nurses who had been working for more than 10 years, and the content of the clinical pathway was reviewed using a Likert 5-point scale, with values of 5, 4, 3, 2, and 1 assigned to Very Important, Important, General, Not Very Important, and Not Important, respectively, and the items with mean scores of 4 or more were retained. The main contents of the revision are as follows: ① No preoperative bowel preparation was revised to enema or oral sodium carbonate salt purgative to clean the bowel 1 day before the operation to avoid contamination of the surgical sterile surface by fecal discharge due to relaxation of the anal sphincter muscle caused by anesthesia. ② The traditional preoperative fasting of 12 and 6 hours was modified to delay fasting until 6 hours preoperatively and fasting until 2 hours preoperatively. Studies have shown that shortening the duration of preoperative fasting and abstinence from food and drink relieves patients’ hunger and thirst, avoids postoperative hypoglycemia, and eases catabolism.^[12]^ ③ Pain management postoperative use of a self-controlled analgesic pump was modified to postoperative administration of pain medication in the epidural space, and the level of pain was assessed using a visual analog scale, with greater than a score of 2 with pain medication (flurbiprofen ester injection, diazoxide, and banco).^[13]^ For oral pain in lingual mucosal graft urethral reconstruction, a visual analog scale was used to assess the level of pain; <2 points were applied to both cheeks with cooling patches, and for >2 points in addition to the cooling patches, 0.5% lidocaine gargle was used in severe cases.^[14]^
3. Act. Theoretical training in the form of PowerPoint and mind mapping was conducted by the researcher in 2 sessions at the noon meeting for all nurses in the department for 30 minutes each time, requiring nurses to master the clinical care pathway and the 1st training focused on the updated clinical care pathway, informing the nurses to go through the evidence-based, and informing the patients preoperatively about the purpose and significance, precautions, and knowledge of the diseases associated with urethral stenosis concerning the urethral breaks, urine cultures, and urinography and urinary flow rate. Patient’s postoperative activities, diet, pain, and psychological care. The 2nd training mainly introduced the common complications of postoperative patients, the critical points of postoperative observation of anterior and posterior urethral stenosis, the necessity and importance of nursing care related to each urethral duct, and the importance of the prevention of urinary tract infection during the perioperative period, and then, the clinical nursing pathway for male urethral stenosis was comprehensively implemented in the department. The researcher and the head nurse followed up. They instructed 1 nurse in charge daily to make him/her care for male urethral stenosis by the clinical nursing pathway and to observe the implementation of all the measures.
4. Observation, reflection, revised plans, and implementation. The clinical care pathway for male urethral stricture operates on a monthly cycle. The existing challenges are systematically reviewed through monthly focus interviews with nurses, leading to the identification of issues. Subsequently, proposed solutions are delineated, and the program undergoes adjustments for progression into the subsequent cycle. After 2 cycles of this study, the primary challenges and corresponding solutions are delineated as follows. The findings from the initial round of nurse-focused interviews revealed that while the current clinical care pathway delineated distinct stages of preoperative and postoperative care, the daily nursing considerations and corresponding measures for each stage lacked uniformity. This incongruence led to a lack of clarity among nurses regarding their daily nursing priorities in comparison to the existing care pathway diagram during the implementation process. Resolution: The nursing considerations and precise nursing interventions for each phase of preoperative and postoperative care were delineated distinctly. Subsequent to the second round of nurse-focused interviews, it was discerned that there were shortcomings in the health promotion measures for patients post-discharge. Resolution: The department proactively disseminated its proprietary “Urethral Stenosis Post-Discharge Health Education Manual” to patients and their families prior to discharge. Post-discharge, a seamless continuum of care was implemented. Consequently, a comprehensive clinical care pathway tailored for male urethral stenosis patients was ultimately established (see Table 1).

### 2.4. Observation indicators

1. WHOQOL-BREF.^[15]^ The WHOQOL-100 Simplified Scale serves as an assessment tool for evaluating the quality of life, derived from the comprehensive WHOQOL-100. Notably, WHOQOL-BREF, a validated instrument tested across 13 countries, has demonstrated equivalence to WHOQOL-100. Professor Fang Jieqian of Sun Yat-sen University of Medical Sciences undertook the revision and translation of the WHOQOL-BREF into Chinese. This instrument is employed for assessing the health-related quality of life for individuals and has gained recognition as a health industry standard sanctioned by the Chinese government. The scale comprises 26 inquiries, with the initial 2 questions dedicated to general assessments, while the subsequent 24 questions encompass 4 dimensions: physical, psychological, social, and environmental aspects. Each query is assessed on a 5-point scale, ranging from a minimum of 1 to a maximum of 5. The physical dimension entails a score ranging from 7 to 35, the psychological dimension ranges from 6 to 30, the social dimension spans from 3 to 15, and the environmental dimension varies from 8 to 40. Importantly, all scores in each domain are positively oriented, signifying that a higher score corresponds to an enhanced quality of life. The Research Group for Quality of Survival at the World Health Organization conducted an analysis of data derived from 20 studies spanning 18 countries. This investigation focused on evaluating the short form’s reliability, validity, and various measurement indicators. The findings underscored the short form’s commendable internal consistency, discriminant validity, and structural validity. The Cronbach alpha coefficient of the scale is 0.863, and the Cronbach alpha coefficients of the 4 domains are all over 0.50.
2. Self-rating Anxiety Scale.^[16]^ Zung introduced the Anxiety Status Scale (SAS) in 1971 as a tool for screening anxiety and assessing the severity of its symptoms. The SAS finds extensive application in clinical settings, facilitating the screening of individuals with or without anxiety symptoms and tracking changes in patients undergoing treatment. The scale comprises 20 items, each evaluated on a 4-point scale: 1 = absence or minimal duration of the symptom; 2 = a brief period with the symptom; 3 = a substantial duration with the symptom; 4 = predominant or continuous presence of the symptom. Fifteen out of the 20 items received negative evaluations, indicated by scores of 1, 2, 3, and 4. Conversely, the remaining 5 items (5, 9, 13, 17, and 19) received positive assessments, with scores of 4, 3, 2, and 1. The summation of scores for all items resulted in a total SAS score. A total SAS score below 50 signified the absence of anxiety, while scores ranging from 50 to 59 denoted mild anxiety, 60 to 69 indicated moderate anxiety, and a score of 70 or higher signified severe anxiety.
3. Self-rating Depression Scale (SDS).^[16]^ Zung introduced this scale in 1965 for the screening and clinical assessment of depression. Comprising 20 declarative sentences with corresponding problem items, each item utilized a 4-level scoring system. The scoring criteria were as follows: 1 point denoted no or minimal time spent with the symptoms; 2 points indicated a brief duration of symptoms; 3 points reflected a substantial period with symptoms; and 4 points represented symptoms persisting most or all of the time. Among these, 10 items received positive scores (1, 2, 3, 4 points successively), whereas items 2, 5, 6, 11, 12, 14, 16, 17, 18, and 20 were assigned reverse scores (4, 3, 2, 1 points successively). The cumulative score is derived from the summation of all individual scores. A total SDS score below 53 signifies a psychologically normal state, while scores ranging from 53 to 62 indicate mild depression, 63 to 72 suggest moderate depression, and scores exceeding 72 represent severe depression.

### 2.5. Data collection and statistical analysis

Prior to the intervention, team members provided a standardized explanation of the questionnaire completion procedure to admitted patients. In cases of dyslexia, team members read the questionnaire aloud, allowing patients to independently respond. The designated time for questionnaire completion was 30 minutes, a duration validated by the researcher, and the completed questionnaires were promptly collected on-site. Following a 3-month intervention, a questionnaire survey was administered during the outpatient reexamination. To uphold questionnaire validity, any questionnaire displaying errors or omissions was deemed invalid.

SPSS 21.0 statistical software was employed for data analysis. Measurement information was expressed as mean ± standard deviation, the *t*-test for normal distribution, the Mann–Whitney *U* nonparametric test for non-normal distribution, and the *x*^2^ test for count data. *P* < .05 was considered a statistically significant difference.

### 2.6. Quality control

1. Following the patients’ voluntary agreement and signing of the informed consent letter, the research team, with collaboration from the urological medical staff, administered a questionnaire survey. To enhance participant engagement, small tokens of appreciation were provided to the survey subjects during the investigation.
2. Preceding the investigation, a comprehensive training session was conducted for the investigators to ensure truthful and objective responses from the research subjects. Standardized language was employed for addressing questions and resolving queries. The investigators were explicitly apprized of the investigation’s purpose and significance.
3. Investigators perform individualized interviews, administering on-the-spot questionnaires that are collected upon completion and scrutinized immediately.
4. The data entry utilized a double-entry method, and any invalid questionnaires were promptly excluded from the dataset.

## 3. Results

### 3.1. Comparison of baseline data between the 2 groups (see Table 2)

The 2 groups of patients were compared in terms of age, education level, payment method, urethral stricture site, and surgical procedure in general.

**Table 2:** Comparison of baseline data between the 2 groups of patients.

	Control subjects n = 45	Test group n = 45	*χ*^2^/*t*	*P* value
Age (yr)	63.08 ± 7.13	62.95 ± 15.02	0.773	.458
Educational attainment			0.304	.859
Junior high school and below	20	19		
High school	18	17		
Undergraduate and above	7	9		
Payment method			0.090	.764
Self-financed	7	6		
Medical insurance	38	39		
Urethral stricture site			0.526	.468
Membrane	32	35		
Globe	13	10		
Surgical procedure				
Urethral reconstruction	18	20	0.843	.656
Urethral stricturotomy	16	12		
Urethral anastomosis	11	13		

### 3.2. Comparison of WHOQOL-BREF scores before and after intervention in both groups (see Table 3)

There was no statistically significant difference between the pre-intervention WHOQOL-BREF scores of all dimensions and the total scores of the 2 groups (*P* > .05), but the post-intervention WHOQOL-BREF scores of all dimensions and the total scores of the 2 groups were compared with each other. The difference was statistically significant (*P* < .05).

**Table 3:** Comparison of WHOQOL-BREF scores before discharge between the 2 groups of patients.

Groups	Precedent	Physiological		Psychosocial		Environments		Social relation		Totals	
		Pre-intervention	Post-intervention	Pre-intervention	Post-intervention	Pre-intervention	Post-intervention	Pre-intervention	Post-intervention	Pre-intervention	Post-intervention
Observation group	45	14.57 ± 3.55	21.26 ± 5.94	6.24 ± 1.11	10.07 ± 1.29	15.09 ± 5.15	23.08 ± 4.21	13.07 ± 3.55	18.06 ± 1.64	48.97 ± 13.36	94.48 ± 16.15
Control subjects	45	14.73 ± 4.02	17.36 ± 5.52	6.35 ± 1.27	8.32 ± 1.08	14.78 ± 4.21	17.59 ± 2.89	12.52 ± 4.02	14.18 ± 1.38	48.38 ± 13.52	76.73 ± 13.01
t		0.545	5.258	0.612	3.158	0.742	6.424	0.545	5.258	0.612	7.325
*P* value		.237	.000	.186	.002	.154	.000	.237	.000	.186	.000

WHOQOL-BREF = the WHO Quality of Life Scale.

### 3.3. Comparison of SAS and SDS scores before and after intervention in the 2 groups (see Table 4)

In comparing SAS and SDS scores between the 2 groups of patients before the intervention, the difference was not statistically significant (*P* > .05). Still, after the intervention, the difference between the 2 groups of patients’ SAS and SDS scores was statistically significant (*P* < .05).

**Table 4:** Comparison of SAS and SDS scores before discharge between the 2 groups of patients.

Groups	Precedent	Apprehensive		Gloomy	
		Pre-intervention	Post-intervention	Pre-intervention	Post-intervention
Observation group	45	62.59 ± 8.54	52.79 ± 7.87	64.57 ± 9.03	56.57 ± 8.62
Control subjects	45	61.82 ± 9.57	58.64 ± 9.93	64.85 ± 9.86	60.78 ± 9.46
*t*		0.782	3.962	0.524	2.974
*P* value		.134	.000	.217	.003

SAS = the Anxiety Status Scale, SDS = Self-rating Depression Scale.

## 4. Discussion

### 4.1. Clinical care pathways based on action research methodology can effectively improve clinical nursing practice

The action research method is a special research method that closely integrates practical problems to be solved and researched. The action research method was initially widely used in education and teaching, and in recent years, it has been gradually applied to clinical nursing with remarkable results. Johannessen^[17]^ designed a series of interventions according to the circular spiral structure of the action research method to alleviate the problem of poor sleep quality of dementia patients with essential oil aromatherapy, and the quality of sleep of the patients improved significantly after the intervention. França et al^[18]^ used an action research approach, guided by a problem-based pedagogy, to provide health education on sexual health to 58 blind people enrolled in a charitable, educational institution, which improved their ability to prevent sexually transmitted infections. A clinical care pathway is a model of care based on a disease or surgical procedure that is updated and refined to suit the realities of the unit based on evidence-based care. This study constructed a clinical care pathway based on an evidence-based foundation in line with the actual situation of our department through the action research method. It explored a new clinical care pathway model for our department.

### 4.2. Building clinical care pathways based on action research methodology can effectively improve the quality of life of male urethral stricture patients

Urethral stenosis is a common disease in urology. Its main clinical manifestations are^[19]^ varying degrees of urinary difficulties, increased resistance to urination, the appearance of a thin urine stream, prolonged urination time, and other phenomena that need to be retained catheter or cystostomy tube to discharge the urine so that their ability to take care of themselves is seriously reduced. They need to rely on their family members too much, and thus, it will make the patient feel a sense of guilt psychology. Sexual dysfunction is the biggest problem faced by patients with urethral stenosis in terms of life,^[20]^ which tends to cause patients to develop a severe inferiority complex and thus lose self-confidence. It has been found that reduced self-confidence is one of the most critical factors affecting patients’ quality of life.^[20]^ The purpose of healthcare is not only to treat the disease itself but also to focus on the patient’s quality of life.

Scholars both domestically and internationally have formulated a range of specialized assessment scales for appraising the quality of life among postoperative individuals with urethral stricture. These scales encompass the Quality of Life Score, WHOQOL-BREF, and the General Quality of Life Questionnaire-74, among others. These instruments are designed to assess the influence of symptoms on daily life, with the WHOQOL-BREF being the most frequently employed. Accordingly, this study opted for the WHOQOL-BREF as the chosen evaluative indicator. Jhanwar et al^[21]^ conducted an inquiry involving 180 individuals afflicted with urethral stricture, revealing a comprehensive postoperative quality of life score of 75.87 ± 15.37. This study, focusing on postoperative patients in the control group, observed a WHOQOL-BREF score of 76.73 ± 13.01, aligning closely with the findings reported by Jhanwar et al The WHOQOL-BREF score during the postoperative phase for patients under observation stood at 94.48 ± 16.15, markedly surpassing that of the control group. This disparity demonstrated statistical significance (*P* < .05). The findings indicate that the implementation of clinical care pathways constructed through the action research method yields a significant enhancement in the quality of life for male urethral stricture patients. Yang’s investigation demonstrated that the clinical care pathway developed for acute myocardial infarction patients using the action research method contributes to the enhancement of patient’s quality of life.^[22]^ Hu et al^[23]^ observed that respiratory training and rehabilitation care, employing the action research method for elderly chronic obstructive pulmonary disease patients, yielded substantial improvements in pulmonary function and quality of life. These findings align with the outcomes of the present study. Table 2 illustrates that, following the intervention with the action research method, the WHOQOL-BREF scores in the observation group exhibited a notable increase compared to the control group, and these differences achieved statistical significance (*P* < .05). The rationale for this lies in the fact that the physiological dimension score predominantly signifies the patient’s recovery status. This study intricately integrates the action research method with the clinical nursing pathway in the clinical nursing practice of male urethral stenosis patients. Throughout the implementation process, patients offer timely feedback based on an evidence-based approach, allowing for prompt issue resolution and continuous enhancements. The perioperative care is systematically addressed by targeting the 3 phases—preoperative, postoperative, and post-discharge. Evidence-based improvements are instituted in various aspects of traditional perioperative care, such as enhanced patient education, scientifically managed preoperative dietary schedules, and improved analgesic care. This approach minimizes perioperative stress, enhances surgical tolerance, and focuses on postoperative measures like reinforcing pelvic floor muscle function exercises to facilitate the recovery of urinary system function. Consequently, the physiological dimension score in the observation group exhibited a significant increase compared to the control group. The social relationship dimension score primarily delineates the interactions between patients and healthcare providers. The clinical nursing pathway formulated in this study delineates meticulous nursing interventions and comprehensive health promotion strategies at each stage, providing valuable guidance for less experienced nurses. This approach empowers nurses to proactively engage in nursing responsibilities beyond mere adherence to physician directives, facilitating the predictable execution of nursing tasks according to the clinical nursing pathway. Patients, in turn, benefit by gaining anticipatory insights into the required actions at each stage, actively participating in their therapeutic and nursing journey and fostering effective communication with medical staff throughout their hospitalization. This proactive engagement contributes to the establishment of robust doctor-patient relationships during the hospitalization period. As a result, the social relationship dimension score and independence dimension in the observation group exhibited significant elevation compared to the control group. In contrast to preceding nursing pathways, the clinical nursing pathway devised in this investigation not only emphasizes the execution of nursing interventions but also places increased emphasis on addressing patients’ psychological well-being. The modes of health education are more diverse, extending the health education environment beyond the hospital setting. The comprehensive health education process enhances patients’ understanding of the disease and mitigates uncertainty regarding their medical condition. Consequently, the psychological dimension and environmental dimension scores of the patients surpassed those of the control group. The foremost factor influencing the emotional state of post-urethral stricture patients is identified as the challenge of urination.^[1]^ Throughout the course of this study, patients received meticulous guidance concerning postoperative urinary function training during both hospitalization and discharge. To augment this, the initiation of a WeChat group was mandated, necessitating the active participation of family members who were tasked with daily supervision and check-ins. Any deviations from prescribed procedures were promptly identified, with immediate guidance provided to rectify irregularities in patient movements. Moreover, the WeChat group consistently disseminates and broadcasts comprehensive information related to urethral stenosis care. This initiative enhances patients’ understanding of the condition, fostering a deeper comprehension of self-care practices. Patients endowed with higher executive capabilities exhibit proficiency in mastering a broader spectrum of self-care behaviors through autonomous endeavors. This self-driven approach not only facilitates the correction of detrimental habits but also enhances compliance with medical interventions. Consequently, these concerted efforts significantly contribute to the restoration of patients’ voiding function, thereby resulting in an improvement in the overall quality of life for the patients.

### 4.3. Building clinical care pathways based on action research methodology can effectively reduce anxiety and depression in male urethral stricture patients

The prevailing psychological challenges encountered postoperatively in male urethral stricture patients primarily manifest as anxiety and depression, exhibiting a documented incidence rate of 58.7%.^[24]^ The etiology of anxiety and depression primarily stems from factors such as limited disease awareness, low health literacy, advanced age, and pronounced somatic symptoms among patients.^[25]^ Patients in both cohorts of this investigation, characterized by an average age surpassing 60 years, exhibited a proclivity towards anxiety and depression. The clinical care pathway, meticulously devised through the action research method in this study, stands out for its comprehensive health education at every stage. This approach enables patients to acquire timely insights into their medical conditions and surgical procedures, fostering a constructive mindset and mitigating their psychological burden in confronting the challenges posed by their ailments. Concurrently, consistent in-person engagement and health education constitute inherent psychological interventions. This holds particular significance for patients dwelling in solitude, those navigating through divorce, or those contending with chronic comorbidities. The recurrent and multifaceted interactions not only supply patients with essential information but also offer a form of emotional support, contributing to the mitigation of negative emotions. Consequently, the observed levels of anxiety and depression in the intervention group were markedly lower than those in the control group.^[26]^

## 5. Conclusions

In conclusion, the establishment of clinical care pathways grounded in the action research method has demonstrated notable efficacy in enhancing both clinical nursing procedures and the overall quality of life for patients, concurrently leading to a reduction in the prevalence of anxiety and depression. Nevertheless, certain constraints must be acknowledged within the scope of this study. The investigation concluded 3 months post-patient discharge, suggesting that future studies could extend the observational period to facilitate a more comprehensive tracking and analysis of outcomes. Additionally, the absence of geographic diversity within the study population, all originating from the same city, underscores the imperative for broader clinical evidence in subsequent research endeavors.

## Author contributions

**Conceptualization:** Xuejing Wang.

**Data curation:** Yanhong Yan.

**Funding acquisition:** Xuejing Wang.

**Formal analysis:** Anqi Li.

**Investigation:** Aiying Yang, Jun Tao.

**Methodology:** Yanhong Yan, Xuejing Wang.

**Project administration:** Xuejing Wang.

**Software:** Yanhong Yan.

**Supervision:** Yanhong Yan.

**Validation:** Yue Wu.

**Writing – original draft:** Yanhong Yan.

**Writing – review & editing:** Yanhong Yan, Xuejing Wang.

## References

[1] Chung PH Leong JY Machado P. Contrast-enhanced ultrasound and shear wave elastography: novel methods for the evaluation of urethral stricture disease. J Urol. 2022;207:152–60. 34428090 10.1097/JU.0000000000002146

[2] Yaméogo CAMKD Mahamat MA Kirakoya B. Male urethral strictures in ouagadougou (Burkina Faso): epidemiological diagnostic and therapeutic aspects. J Urol (English). 2020;10:101–10.

[3] Abramowitz D Sam AP Pachorek M. Multi-institutional review of non-hypospadiac penile urethral stricture management and outcomes. Int J Urol. 2022;29:376–82. 35118726 10.1111/iju.14786

[4] Kluth LA Dahlem R Becker A. Psychometric validation of a German language version of a PROM for urethral stricture surgery and preliminary testing of supplementary ED and UI constructs. World J Urol. 2016;34:369–75. 26049865 10.1007/s00345-015-1610-8

[5] Sütdelen E Haberal HB Guliyev F. Urethral stricture is an unpleasant complication after prostate surgery: a critical review of current literature. J Urol Surg. 2016;3:1–6.

[6] Belbina SH Spivey-Provencio S Taheri P. Robot-assisted laparoscopic prostatectomy and urethral pull through of membranous urethra to bladder. Videourology. 2022;36:7–7.

[7] Fan Z Jing-Ping Z. Construction of ICU nursing monitoring and evaluation scale for multiple trauma patients based on delphi-AHP method. J Nurs (China). 2019;26:11–5.

[8] Moch SD Vandenbark RT Pehler SR. Use of action research in nursing education. Nurs Res Pract. 2016;2016:8749167. 28078138 10.1155/2016/8749167 PMC5203904

[9] Webb C. Action research: philosophy, methods and personal experiences. J Adv Nurs. 1989;14:403–10. 2738236 10.1111/j.1365-2648.1989.tb01548.x

[10] Jiaojiao WU Hongmei MA Chunxia L. The application status of medical integration working model in the nursing field. China Med Her. 2017;14:38–42.

[11] Bhatt S Goel M Gupta A. Diagnosis of urethral stricture on dynamic voiding transvaginal sonourethrography: a case report. J Diagn Med Sonography. 2017;33:140–3.

[12] Feldheiser A Aziz O Baldini G. Enhanced Recovery After Surgery (ERAS) for gastrointestinal surgery, part 2: consensus statement for anaesthesia practice. Acta Anaesth Scand. 2016;60:289–334. 26514824 10.1111/aas.12651 PMC5061107

[13] Shun-Hua D Hui-Xia M Yin-Lian L. The effect observation of application of the clinical nursing pathway in patients undergoing transurethral resection of prostate. Guide China Med. 2009;7:5–7.

[14] Vissink A Burlage FR Spijkervet FKL. Prevention and treatment of salivary gland hypofunction related to head and neck radiation therapy and chemotherapy. Supportive Cancer Ther. 2004;1:111–8. 10.3816/SCT.2004.n.004 18628187

[15] Bonomi AE Patrick DL Bushnell DM. Validation of the United States’ version of the World Health Organization Quality of Life (WHOQOL) instrument. J Clin Epidemiol. 2000;53:1–12. 10693897 10.1016/s0895-4356(99)00123-7

[16] Korkidakis A Bryson P Jamieson MA. Outcomes of a decade of routine cervical screening in a Canadian adolescent obstetrics clinic. J Obstet Gynaecol Can. 2016;38:51–5. 26872756 10.1016/j.jogc.2015.12.001

[17] Johannessen B. Nurses experience of aromatherapy use with dementia patients experiencing disturbed sleep patterns. An action research project. Complement Ther Clin Pract. 2013;19:209–13. 24199975 10.1016/j.ctcp.2013.01.003

[18] França ISX Coura AS Sousa FS. Acquiring of knowledge about sexual health by blind people: an action research. Rev Lat Am Enfermagem. 2019;27:e3163. 31340347 10.1590/1518-8345.3006.3163 PMC6687357

[19] Benson CR Brandes SB. Contrast-enhanced ultrasound and shear wave elastography: novel methods for the evaluation of urethral stricture disease letter. J Urol. 2022;208:563–4. 35648645 10.1097/JU.0000000000002786

[20] González-Espinosa C Castro-Nuez P Averbeck MA. Diagnosis and treatment of urethral stricture in men with neurogenic lower urinary tract dysfunction: a systematic review. Neurourol Urodyn. 2022;41:1248–57. 35686544 10.1002/nau.24982

[21] Jhanwar A Sokhal AK Singh K. Assessment of quality of life in patients of urethral stricture on clean intermittent catheterization following direct vision internal urethrotomy. Urol Ann. 2018;10:395–9. 30386093 10.4103/UA.UA_34_17 PMC6194795

[22] Yang SN. Construction and application effects evaluation of clinical nursing pathway for patients with acute myocardial infarction based on action research. Chin Nurs Res. 2021;35:4265–9.

[23] Hu ZJ He J Wang M. Effect of respiratory training and rehabilitation nursing based on action research on lung function and quality of life in elderly patients with chronic obstructive pulmonary disease. J Baotou Med. 2023;47:57–9.

[24] Barbalias D Lappas G Ravazoula P. Evaluation of the distribution of paclitaxel after application of a paclitaxel-coated balloon in the rabbit urethra. J Endourol. 2018;32:381–6. 29382215 10.1089/end.2017.0935

[25] Xia MH Liu JL Hao N. Clinical study on long-snake moxibustion plus Western medicine in treating chronic heart failure due to heart-kidney yang deficiency. J Acupunct Tuina Sci. 2021;19:284–90.

[26] Niu C Huang X Wang L. Effect of hospital, community and home care model on nursing and quality of life of patients after transurethral resection of benign prostatic hyperplasia. Am J Transl Res. 2021;13:4959–68. 34150080 PMC8205839

